# Sleep quality in relation to trait energy and fatigue: an exploratory study of healthy young adults

**DOI:** 10.5935/1984-0063.20210002

**Published:** 2022

**Authors:** Nicholas Pastier, Erica Jansen, Ali Boolani

**Affiliations:** 1 Clarkson University, Physical Therapy - Potsdam - New York - United States.; 2 University of Michigan, Nutritional Science - Ann Arbor - Michigan - United States.

**Keywords:** Fatigue, Sleep, Affect

## Abstract

**Objectives:**

To evaluate how sleep quality predicts the trait aspect of mental and physical energy versus fatigue within a healthy non-obese adult population.

**Material and Methods:**

A study population of 495 adults completed an online survey concerning trait energy and fatigue as well as sleep quality. Bivariate and adjusted analysis examined whether sleep quality, measured from the Pittsburgh sleep quality instrument, associated with physical and mental trait energy and fatigue (4 separate constructs). Adjusted analysis included caffeine consumption, polyphenol consumption, current mood, perceived mental workload, and physical activity.

**Results:**

Bivariate analysis showed that both physical and mental fatigue were associated with sleep quality, while physical and mental energy were not. However, after adjustment for potential confounders, sleep quality was associated with mental fatigue and physical energy (not physical fatigue).

**Conclusion:**

Findings suggest that improvement in sleep quality among healthy young adults may affect certain aspects of physical versus mental energy and fatigue more strongly than others.

## INTRODUCTION

Adequate sleep quality is important for achieving overall health and high quality of life. While multiple studies have reported the effects of poor sleep quality on transient (state) feelings of energy and fatigue^1-3^, the relationship between the long-standing pre-disposition (trait) energy and fatigue and sleep quality remains largely unexplored. Trait energy and fatigue are often assumed to be part of the same construct, with high energy at one end and high fatigue at the other end. However, recent studies show that these constructs are likely distinct, given that they have different predictors and also relate differently to health outcomes^1,4-6^. Another distinction is physical versus mental energy and fatigue^4,7^. Yet, how sleep quality relates to each of these constructs remains unclear, particularly within otherwise healthy young adult cohorts. We hypothesized that sleep quality may be more highly predictive of certain aspects of trait mental and physical energy and fatigue than others (a specific hypothesis about the strongest relationship was not made due to lack of prior literature).

## MATERIAL AND METHODS

Approval for this study was granted by Clarkson University Institution Review Board (Approval #16-34.1). Participants were recruited from a small, engineering university and the surrounding town (approximately 16,000 total population), and subjects provided documented consent within the internet-based survey. A detailed description of this study has been previously published (Boolani and Manierre (2020))^4^. From the 1,007 participants who attempted the survey, we eliminated 249 for incomplete responses, 2 for missing data, 96 for chronic diseases, 29 for being smokers, 29 for being under the age of 18 or over the age of 45, and 78 for reporting a BMI <18.5 or >30.0. After removal of outliers, as outlined in the analysis section, we had a usable sample of 495 participants.

### Instruments

**Sleep quality:** to assess global sleep quality score, we used the Pittsburgh sleep quality inventory. The 19-question survey assesses seven dimensions of sleep: sleep quality, latency, duration, habitual sleep efficiency, sleep disturbances, use of sleep disturbances, and daytime dysfunction over the past month. The components scores were summed and reported as global sleep quality index, where higher scores indicate worse sleep quality^8^. In our current study, we also sub-divided our participants into good sleepers (PSQI scores <5) and bad sleepers (PSQI score ≥5)^8^.

### Trait moods

The mental and physical energy and fatigue trait-state scale was used to assess trait mental and physical energy^9^. The three-part scale measures mental and physical energy and fatigue scale and traits. For the purposes of this study, we only used the trait aspect of this scale. The trait aspect is a 12-item measure using a Likert scale (0=never to 4=always) to measure the presence of disposition of mental and physical energy and fatigue. Participants are asked about their usual feelings of a synonym (e.g., “I usually feel full of pep”) and are asked to rate themselves on the 0 to 4 scale. Three synonyms are added together to form a trait. Among healthy adults, the Cronbach’s alpha coefficients range from .82 to .91^9^ and in the current data the alphas ranged from .82 to .93 (trait physical energy=.82, trait physical fatigue=.90, trait mental energy=.86, and trait mental fatigue=.93).

### Covariates

As potential confounders, we assessed caffeine consumption, polyphenol consumption, current mood, perceived mental workload, and physical activity. Caffeine was estimated by asking participants a series of questions regarding their consumption of 89 different caffeine containing foods and beverages^10^. Polyphenols have been known to positively influence sleep^11^, so consumption was estimated using a food frequency questionnaire^12^ of 56 polyphenol dense foods (i.e., fruits, vegetables, beverages containing polyphenols, and chocolate)^12^. The final scale reflects servings of individual polyphenols per month.

The profile of mood survey-short form (POMS-SF) was used to measure six different dimensions of state moods over the last 30 days. Participants were asked to assess their moods on a five-point scale ranging from “not at all” (scored as 0) to “extremely” (scored as 4) on 30 different synonyms associated with the different mood states (e.g., full of pep, vigorous for energy)^13^. Feelings of fatigue (α=.84), energy (α=.85), tension/anxiety (α=.81), anger (α=.82), and depression (α=.94) were measured on a 0 to 20 scale. Confusion (α=.48) was measured on a-4 to 16 scale. Perceived mental workload, which incorporates both time spent doing mental work as well as intensity, on both work days and non-work days was calculated using the mental and physical energy and fatigue trait-state scale, in a manner consistent with prior research^1,14^ and the manual^9^. Using the first part of the mental and physical energy and fatigue trait-state scale, metabolic equivalents (METS) were calculated for high and moderate physical activity. Consistent with the manual^9^ and prior literature^10^, participants were asked to identify the number of hours per day and the number of days per week that they participated in moderate and vigorous physical activity. Results are reported in METS/week.

### Statistical analyses

Participants who reported scores >3 SD away from the mean on any of the reported measures were removed. Independent sample *t*-tests were used to test for differences between lifestyle factors and trait and state moods between good and bad sleepers. Chi-square tests were performed to test for sex differences between good and bad sleepers. A multivariate multiple regression was used to examine the association between trait fatigue and energy and sleep quality.

All analyses were conducted using IBM SPSS Statistics for Mac (IBM Corp, Version 26.0 Armonk, NY, U.S.). For all analyses in this study our α was set at 0.05. A post hoc power analysis was completed for both F-family and T-family tests using G*Power (version 3.1.9.2)^15^.

## RESULTS

### Predictors of poor sleep quality

In bivariate analysis, those with poor quality sleep reported feeling significantly more fatigued (*p*<.001), confused (*p*<.001), tense/anxious (*p*<.001), depressed (*p*=.001), angry (*p*=.001), and less energetic (*p*=.002). Overall, those with poor quality sleep had higher total mood disturbance (*p*<.001) compared to those with high quality sleep. Poor sleep quality was also associated with drinking more servings of coffee (*p*=.001) and more servings of energy beverages (*p*<.001), and overall caffeine per week (*p*<.001). Poor sleep quality was related to consuming more vegetables (*p*=.008) and beverages containing polyphenols (*p*<.001); however, there was no significant difference in total polyphenol consumption (*p*=.107). Those with poor sleep quality also reported higher trait physical (*p*=.032) and mental fatigue (*p*=.001) than good sleepers. No other variables were statistically significant (*p*>.05) (Table 1).

**Table 1:** Comparing participants with good and poor quality sleep.

Variables	Participants with good quality sleep (n=247)	Participants with poor quality sleep (n=243)	F-value	*p*-value
Age	20.66 ± 2.98	20.48 ± 3.215	0.20	0.66
Sex Male Female	63.6% 36.4%	62.6% 37.4%	0.001	0.973
Body mass index (BMI)	22.99 ± 2.61	23.24 ± 2.77	0.82	0.37
**Moods**				
POMS Vigor	9.51 ± 4.31	8.36 ± 3.92	1.69	0.20
POMS Fatigue	3.33 ± 2.76	5.45 ± 3.75	27.84	0.00
POMS Confusion	-0.58 ± 1.87	0.20 ± 2.25	4.67	0.03
POMS Tension	2.22 ± 2.59	3.27 ± 2.93	9.61	0.00
POMS Depression	1.43 ± 2.30	2.26 ± 2.77	9.18	0.00
POMS Anger	1.49 ± 2.11	2.22 ± 2.66	9.30	0.00
POMS Total Mood Disturbance	-1.62 ± 10.25	5.05 ± 11.66	7.04	0.01
**Caffeine consumption per week**				
Coffee	4.62 ± 5.20	6.33 ± 6.48	10.65	0.00
Tea	3.26 ± 5.04	4.06 ± 5.56	1.73	0.19
Soda	3.90 ± 5.34	4.38 ± 6.19	2.89	0.09
Energy beverages	0.92 ± 2.09	1.86 ± 3.52	38.88	0.00
Cocoa containing caffeine	0.96 ± 2.20	0.83 ± 2.32	0.50	0.48
Over the counter medication	0.04 ± 0.38	0.04 ± 0.32	0.00	0.98
Total servings of caffeine	13.79 ± 9.66	17.58 ± 12.34	7.22	0.01
**Polyphenols besides caffeine consumption per month**				
Fruit	18.03 ± 17.47	19.64 ± 19.97	0.55	0.46
Vegetable	38.12 ± 27.20	45.42 ± 37.71	7.09	0.01
Beverages containing polyphenols	10.95 ± 9.70	13.44 ± 12.07	15.12	0.00
Chocolate	9.21 ± 9.78	11.14 ± 12.58	5.24	0.02
All other polyphenols	76.31 ± 46.55	89.63 ± 61.30	2.61	0.11
**Mental workload**				
Work-day mental intensity	109.59 ± 67.98	119.00 ± 65.63	0.01	0.95
Off-day mental intensity	20.78 ± 20.61	20.37 ± 17.18	1.90	0.17
**Trait moods**				
Trait physical energy	7.07 ± 2.08	6.37 ± 2.09	0.43	0.51
Trait physical fatigue	3.49 ± 1.84	4.37 ± 2.12	4.64	0.03
Trait mental energy	6.32 ± 2.07	5.49 ± 2.04	0.35	0.55
Trait mental fatigue	3.55 ± 1.86	5.19 ± 2.24	10.73	0.00
Physical activity				
High intensity physical activity	48.28 ± 41.55	53.66 ± 40.95	0.23	0.63
Moderate intensity physical activity	5.90 ± 7.48	5.78 ± 6.57	0.01	0.94
Physical activity composite score	211.45 ± 34.77	215.52 ± 33.65	0.11	0.74

The multiple linear regression accounted for 22.6% of the variation in PSQI global sleep quality scores (R^2^=.256, F(19, 470)=8.526, *p*<.001). After accounting for all potential confounders, sleep quality was statistically significantly associated with lower trait physical energy (β=-.168, t(470)=-3.084, *p*=.002) and higher trait mental fatigue (β=.329, t(470)=5.810, *p*<.001), but not with physical fatigue or mental energy.

## DISCUSSION

This study provides us with some interesting insights into the relationship between trait energy and fatigue, and sleep quality with a relatively young and non-obese population. Overall, the strongest relationship was between trait mental fatigue and sleep quality. Our unadjusted results indicated that participants who reported poor sleep quality also reported higher trait physical and mental fatigue but not lower trait mental and physical energy. Similarly, state fatigue (i.e., current mood) was associated with worse sleep quality, but state energy was not. These findings are of interest because it continues to support recent evidence that energy and fatigue are distinct moods^1,5,6,16^. Although the concept of trait fatigue as a separate and unique trait from trait energy is new, the proposed mechanisms of fatigue by Loy et al. (2018)^16^ are some of the same mechanisms that have been associated with poor sleep quality such as inflammatory cytokines,^17^, serotonin^18^, and histamine.^19^

In the adjusted analysis, poorer sleep quality was associated with lower trait physical energy; however, poorer sleepers did not have lower trait mental energy. There could be a specific mechanism to explain this finding. Dopamine, a neurotransmitter, has been associated with influencing sleep^20^ and feelings of energy^16^. Boolani et al. (ANO)^1^ report that increased feelings of energy were associated with improved skeletal muscle mitochondrial function, while poor sleep has been associated with skeletal muscle mitochondrial dysfunction^21^.

This study has several limitations, including the use of self-reported measures and the fact that we did not ask about the timing of caffeine or polyphenol intake. Poor sleep quality was associated with drinking more servings of coffee and energy beverages, and overall caffeine per week, but the utility of those results is limited because of the self-reported nature of those measures and the fact that we did not ask about the timing of caffeine intake. The cross-sectional design also limits the determination of the causality of the relationship between the variables and sleep quality.

Ultimately, these findings suggest that sleep quality must be considered when trying to understand ways to improve the long-standing pre-disposition to energy and fatigue in young healthy adults. Although this study did not measure underlying biological or genetic components associated with poor sleep quality, the results from this study suggest that the need to examine biological and/or genetic components that may influence the long-standing pre-disposition to low physical energy, high mental fatigue, and sleep simultaneously. Healthy young adults who are struggling with habitual low physical energy or with high mental fatigue could benefit from improved sleep quality.

## Figures and Tables

**Table 2:** Predictors of global sleep quality scores.

Variables	B**	t-statistic	p-value	95% CI of b
Trait physical energy	-0.168	-3.084	0.002	(-0.333,-0.074)
Trait physical fatigue	-0.093	-1.682	0.093	(-0.254, 0.020)
Trait mental energy	0.021	0.383	0.702	(-0.104, 0.155)
Trait mental fatigue	0.329	5.890	<0.001	(0.253, 0.506)
Moderate intensity PA	0.021	0.497	0.619	(-0.022, 0.038)
High Intensity PA	0.029	0.636	0.525	(-0.004, 0.007)
All other polyphenols	0.062	1.406	0.160	(-0.001, 0.007)
Number of servings of caffeine/week	0.108	2.557	0.011	(0.006, 0.043)
POMS vigor	-0.044	-0.890	0.374	(-0.086, 0.032)
POMS fatigue	0.213	3.786	<0.001	(0.076, 0.240)
POMS confusion	-0.014	-0.272	0.786	(-0.143, 0.108)
POMS tension	0.061	1.064	0.288	(-0.047, 0.158)
POMS depression	0.061	1.020	0.308	(-0.056, 0.177)
POMS anger	-0.113	-2.032	0.043	(-0.236,-0.004)
BMI	0.017	0.408	0.683	(-0.060, 0.092)
Age	-0.043	-1.007	0.315	(-0.104, 0.033)
Sex	0.055	1.308	0.191	(-0.146, 0.729)
Work-day mental work	-0.031	-0.724	0.470	(-0.004,0.002)
Off-day mental work	-0.027	-0.634	0.527	(-0.015, 0.008)

** B = Standardized coefficient;

* PA = Physical activity.
